# Identification and Containment of a Cluster of Two *Bacillus cereus* Infections in a Neonatal Intensive Care Unit

**DOI:** 10.1155/2019/1506583

**Published:** 2019-01-20

**Authors:** Cihan Papan, Kai Förster, Reinhard Herterich, Andreas Schulze, Sören Schubert, Andreas W. Flemmer

**Affiliations:** ^1^Div. Neonatology, University Children's Hospital and Perinatal Center, Ludwig Maximilian University Munich, Munich, Germany; ^2^Pediatric Infectious Diseases, Medical Faculty Mannheim, Heidelberg University, Mannheim, Germany; ^3^Children's Hospital Landshut, Landshut, Germany; ^4^Max-von-Pettenkofer-Institute Munich, Ludwig Maximilian University Munich, Germany

## Abstract

We report a cluster of invasive *Bacillus cereus* infections in a neonatal intensive care unit. We describe the clinical course of two infected patients, one of whom died of severe pneumonia after successfully being weaned from ECMO. Environmental analyses failed to yield a common source. Molecular characterization confirmed the homogeneity of both isolates. Rigorous hygiene control and adequate therapy enabled the containment of the cluster.

## 1. Introduction

Progress in neonatal medicine has improved the outcome for prematurely born children. Still, mortality and morbidity remain high. Due to the immaturity of their immune system, premature newborns are particularly susceptible to severe infections. Outbreaks especially in the setting of a neonatal intensive care unit (NICU) gained strong media attention [1] in the past years. An outbreak is defined by the World Health Organization as an “unexpected increase in cases” [2] and by the German Infectious Diseases Protection Act and the Robert Koch Institute (the German Center for Disease Control and Prevention) as “at least 2 infected patients linked in time and space” [3]. The majority of publications focused on larger outbreaks of common nosocomial pathogens, such as *Staphylococcus aureus*, or Gram-negative bacteria such as *Klebsiella pneumoniae* [4], while some ubiquitously distributed bacteria are probably underestimated. One such underestimated microorganism is *Bacillus cereus*, a Gram-positive, rod-shaped, spore-building bacterium, closely related to *Bacillus anthracis* and encountered ubiquitously. Its spores are capable of overcoming extreme conditions such as drought, radiation, freeze, or heat. An arsenal of exoenzymes and toxins confers pathogenicity, such as hemolysins, phospholipases, emesis-inducing toxins, and several enterotoxins [5]. *B. cereus* can colonize the gastrointestinal (GI) tract of humans but is also able to cause GI tract infection or food poisoning [5]. Previously, *B. cereus* was mostly regarded as contaminating patient samples with little or no relevance. In recent years, several cases of clinically relevant *B. cereus* infections in neonates were reported, underlining the medical importance of this bacterium in immunocompromised hosts [6]. Neonatal *B. cereus* infections, such as sepsis [7], pneumonia [8], meningitis or meningoencephalitis [9], and enterocolitis [10], have high case-fatality rates [11] and possibly relevant long-term sequelae [12]. Other susceptible cohorts are, e.g., children with oncologic diseases [13], whereas in rare cases, infections have occurred in seemingly immunocompetent individuals [14]. Here, we report a cluster in an NICU involving two patients with invasive *B. cereus* infections.

## 2. Methods

### 2.1. Study Design and Patients

We performed a retrospective study to analyze a cluster of *B. cereus* infections within the setting of an NICU. During the cluster period, a total of 12 patients were hospitalized. A positive case was defined as a *B. cereus* finding in a clinically indicated or surveillance specimen.

### 2.2. Setting

**Figure 1:**
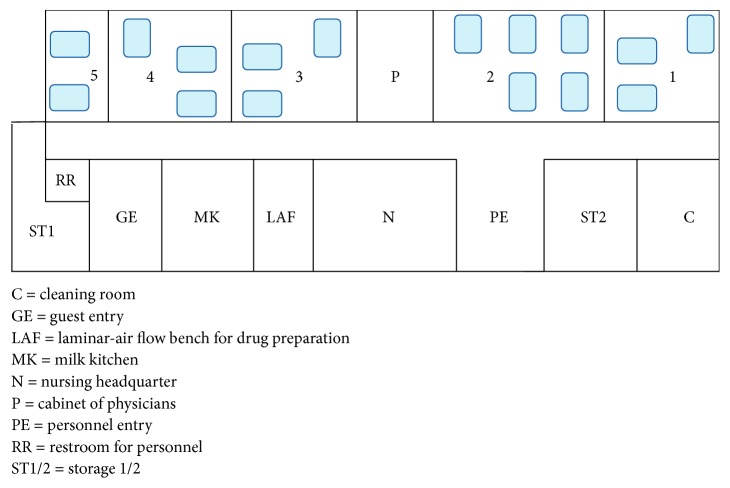
Schematic plan of the neonatal intensive care unit.

The NICU at the University Hospital Ludwig Maximilian University Munich, Germany, is a level-3 unit with 16 intensive care beds (Figure 1) specialized in the care for extremely preterm infants and infants with severe respiratory disease. It is equipped to provide extracorporeal membrane oxygenation (ECMO) therapy and is affiliated to a large perinatal center with approximately 4000 births a year. All patients in the unit are routinely examined once a week for colonization by pathogenic, multiresistant bacteria by taking swabs from nasopharynx, inguinal, and rectal skin.

### 2.3. Microbiological and Molecular Investigations

Microbiological specimens were taken from the affected patients and their environment. From all other patients hospitalized at the time, surveillance swabs were collected twice weekly as described above. In order to quantify the risk of *B. cereus* colonization before and after the cluster, surveillance records of all patients treated at the NICU during 3.5 years prior and 3.5 years after the cluster were reviewed. Positive findings were identified by culture and subsequent matrix-assisted laser desorption/ionization time-of-flight mass spectrometry (MALDI-TOF MS) (Bruker LT Microflex MALDI-TOF MS and Bruker Biotyper 3.0 system software, Bruker Daltonics, Bremen, Germany). Antimicrobial susceptibility testing (AST) included disk-diffusion system and minimum inhibitory concentration (MIC) determination using Etest system (bioMérieux, Nürtingen, Germany). To further analyze detected pathogens, random amplified polymorphic DNA polymerase chain reaction (RAPD-PCR) was performed.

## 3. Results

### 3.1. Report of the Cases

**Figure 2:**
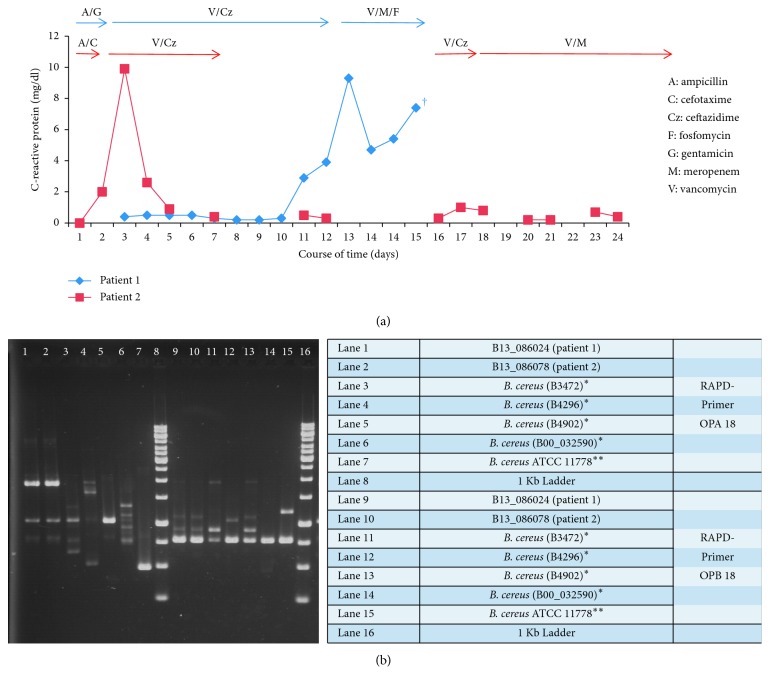
(a) C-reactive protein for both patients, over time and respective antibiotic therapy; (b) *Bacillus cereus* molecular strain typing by RAPD-PCR. ^*∗*^*B. cereus* control strains from the clinical specimen; ^*∗∗*^*B. cereus* ATCC type strain as further control.

Patient #1 (P#1) was born at 37 5/7 weeks of gestation (birth weight 2700 g) and transferred to our hospital on the fifth day of life (DOL) for extracorporeal membrane oxygenation (ECMO) after congenital diaphragmatic hernia closure. Upon arrival, ceftazidime, vancomycin, and fluconazole were started. ECMO was maintained for five days, after which the respiratory situation stabilized. On the third day after being removed from ECMO, the endotracheal tube had to be replaced due to obstruction. The patient showed more desaturations, increased endotracheal secretion, and a higher need of oxygen and iNO. Serum levels of C-reactive protein (CRP) and interleukin-6 were elevated (Figure 2(a)). Chest X-ray revealed patchy consolidations. Blood culture yielded Gram-positive rods, further identified as *Bacillus cereus*. According to the AST result, antibiotic therapy was switched to meropenem, vancomycin, and fosfomycin. The cardiorespiratory situation worsened; vasopressor therapy and intensified ventilation were required. A second ECMO was discussed with the parents but rejected due to the overall poor prognosis. Thus, palliative care was initiated on the eighth day after ECMO, and the patient died. Autopsy revealed pyogenic tracheobronchitis, necrotizing bronchiolitis and pneumonia, with diffuse alveolar desquamations.

Patient #2 (P#2) was born two days after P#1 at 27 1/7 weeks of gestation (birth weight 815 g) due to chorioamnionitis and was initially supported with noninvasive ventilation. Due to increasing FiO_2_, the patient was intubated and underwent surfactant application on the first DOL and later received dexamethasone over a two-day period before extubation was performed on the seventh DOL. On the 17th DOL, the patient presented with a fever of 38.4°C and increasing FiO_2_. Chest X-ray revealed increased bilateral shadowing. The patient had to be intubated again. Vancomycin and ceftazidime were initiated (Figure 2(a)). Microbiological work-up yielded *Bacillus cereus* with low count in the tracheal aspirate. Blood cultures remained sterile. According to the AST result, the therapy was switched to vancomycin and meropenem and continued for 15 days. No further complications occurred.

### 3.2. Environmental Investigations

There were no changes concerning staff or procedural standards prior to the cluster. No other patient was colonized or infected by *B. cereus* during this time. The analysis of the patients' environment, which included the microbiological work-up of milk powder, parenteral nutrition, and medication of P#1 as well as breast milk of P#2's mother failed to detect *B. cereus*. In the analysis of the time before and after the cluster, no cases of *B*. *cereus* infection or colonization in the NICU were detected.

### 3.3. Microbiological and Molecular Investigations

Positive blood cultures (Bactec, BD, Heidelberg, Germany) as well as respiratory specimen and swabs were streaked out on Columbia agar plates on chocolate agar. Bacterial colonies grown over night showed strong hemolysis. The isolates were characterized phenotypically as facultative anaerobic, endospore-forming, gram-positive rods. Identification by MALDI-TOF MS revealed *Bacillus cereus* in both cases.

Both isolates showed good susceptibility to imipenem, meropenem, vancomycin, linezolid, and levofloxacin applying the PK/PD (nonspecies related) breakpoints from the EUCAST breakpoint tables for interpretation of MICs and zone diameters version 4.0. To determine whether the two cases were caused by an identical strain, the isolates were genotyped using RAPD-PCR analysis as described previously [15]. For the RAPD analysis, two primers (OPA 18, OPB 18) generated qualitative RAPD profiles. The strains isolated from both patients were identical and distinguishable from control strains obtained from other patients and from the ATCC 11778 type strain (Figure 2(b)).

### 3.4. Infection Control Assessment

Patient and environmental screening were implemented. Both patients were isolated, and the use of aprons and gloves was ordered. Review of hand hygiene practices among nursing staff and physicians did not identify breeches. Yet, reeducation was enforced. For surfaces, disinfectants effective against spores were used.

## 4. Discussion

We report a cluster of *B. cereus* infections in two neonates, linked in time, space, and strain, aggravating a pulmonary hypertension with a fatal outcome in one of the two newborns.

Our report is limited by the lack of an identified source. Yet, *B. cereus* spores are ubiquitous, and no NICU can be regarded as sterile since parents are involved in the daily care and bonding with their children, e.g., by kangaroo care. This may create a different profile of microbial exposure, in contrast to adult intensive care units. Studies have shown that microbial transfer occurs from parents to the children during intense skin-to-skin contact, but also from inanimate hospital environments to infants, suggesting transfer via the parents' or healthcare workers' hands [16, 17].

Even though there was no immediate proximity, P#2 in room 3 became symptomatic six days after disease onset in P#1 in room 4 and only one day after P#1 had deceased, suggesting a horizontal transfer through the medical staff. Since parents are offered shared rooms in close vicinity to the NICU, one could also speculate on an interparental transfer of pathogens. Furthermore, it is remarkable yet elusive how P#1 could be infected despite sufficient vancomycin levels, but the presence of several risk factors for a severe course rendered both patients especially vulnerable: undergoing ECMO and an organ specific susceptibility (lung hypoplasia and ventilator-induced lung injury) for P#1, prematurity and corticosteroid therapy for P#2, and the presence of invasive devices for both. Remarkably, the pathogen was found in a sterile compartment in P#1, but only in the tracheal aspirate in P#2, which could have been possibly contaminated. Taking the clinical and laboratory findings into consideration, and in knowledge of P#1, this result was appreciated as clinically relevant. However, it must be considered that the respiratory worsening could have been due to the inflammatory response of the premature lungs, which is typically encountered at the 10th to 14th DOL. Nevertheless, both effects could also have taken place simultaneously.

Despite increasing reports in recent years, *B. cereus* has gotten little attention. Schwab et al. analyzed data from the German national nosocomial infection surveillance system for neonatology and estimated 26 to 61 outbreaks to occur annually in German NICUs [18]. Most outbreaks in their analysis were caused by *Staphylococcus aureus* and *Enterococcus* spp., followed by different Gram-negative bacteria and *Candida albicans*. However, none of these outbreaks was linked to *B. cereus*. In an older analysis utilizing an outbreak database, outbreak-database.com, Gastmeier et al. showed that the source of infections remains elusive in almost half of all NICU outbreaks [19]. Outbreaks caused by *B. cereus* are rare, but possibly underreported, due to the notion of its insignificance. Also, the decision to publish or withhold data may be influenced by small sample sizes, the lack of an identified source, or the fear of negative publicity [19].

Awareness has to be raised for the role of *B. cereus* in immunocompromised hosts. Gram-positive rods in sterile compartments, also in airways with a matching clinical picture—distinct from the early-onset neonatal listeriosis—should be used as an indication in order to adapt infection control measures and reevaluate therapy with respect to a possible *B. cereus* infection.

## Data Availability

The data used to support the findings of this study are included within the article.
